# CSF Proteomic Profiles Associated With White Matter Integrity in Cognitively Normal Older Adults With and Without Amyloid Pathology

**DOI:** 10.1212/WNL.0000000000214675

**Published:** 2026-03-02

**Authors:** Luigi Lorenzini, Mario Tranfa, Mara Ten Kate, Anouk den Braber, Lisa Vermunt, Flora H. Duits, Senne B. Lageman, Diederick de Leeuw, Charlotte E. Teunissen, Sara Garbarino, Luca Roccatagliata, Matteo Pardini, Wiesje M. Van Der Flier, Pieter Jelle Visser, Frederik Barkhof, Betty M. Tijms

**Affiliations:** 1 Department of Radiology and Nuclear Medicine, Amsterdam University Medical Centre, Vrije Universiteit, the Netherlands;; 2 Amsterdam Neuroscience, Brain Imaging, the Netherlands;; 3 Department of Neuroscience, Rehabilitation, Ophthalmology, Genetics, Maternal and Child Health (DiNOGMI), University of Genoa, Italy;; 4 Department of Advanced Biomedical Sciences, University of Naples “Federico II”, Italy;; 5 Alzheimer Center Amsterdam, Department of Neurology, Amsterdam UMC, the Netherlands;; 6 Department of Biological Psychology, Vrije Universiteit Amsterdam, the Netherlands;; 7 Amsterdam Neuroscience, Neurodegeneration, the Netherlands;; 8 Neurochemistry Laboratory, Department of Laboratory Medicine, Amsterdam Neuroscience, VU University Medical Center, Amsterdam UMC, the Netherlands;; 9 Neurophysiology and MEG Center, Department of Neurology, Amsterdam Neuroscience, Vrije Universiteit Amsterdam, the Netherlands;; 10 Dipartimento di Matematica (DIMA), Università di Genova, Italy;; 11 IRCCS Ospedale Policlinico San Martino, Genoa, Italy;; 12 Department of Health Sciences (DISSAL), University of Genoa, Italy;; 13 Department of Neuroradiology, IRCCS Ospedale Policlinico San Martino, Genoa, Italy;; 14 Department of Epidemiology & Biostatistics, Amsterdam Neuroscience, Amsterdam UMC, the Netherlands;; 15 Department of Psychiatry, Maastricht University, the Netherlands; and; 16 Institutes of Neurology and Healthcare Engineering, University College London, United Kingdom.

## Abstract

**Background and Objectives:**

Increasing evidence indicates a potential role of white matter (WM) damage in the onset and progression of Alzheimer disease (AD). However, the biological processes underlying in vivo WM imaging biomarkers remain unclear. We sought to determine the molecular signatures associated with WM integrity in cognitively normal individuals with and without amyloid pathology.

**Methods:**

We selected older individuals without dementia (Clinical Dementia Rating <1) from the Alzheimer Centrum Amsterdam when they had diffusion tensor imaging (DTI) and CSF proteomic (untargeted tandem mass-mass spec) data available. Fractional anisotropy (FA) and mean diffusivity (MD) values were computed for the total WM and for 12 tracts of interest. We tested associations between protein levels (predictors) and both global and regional FA and MD values (outcomes) with linear models. Models further included an interaction between protein levels and amyloid status to evaluate specificity to disease. Gene-set and cell-type enrichment analyses were performed on proteins showing significant associations to characterize the underlying biological and cellular processes.

**Results:**

A total of 96 participants were included in this study (mean age 67.82 ± 6.93 years; 45% male participants). A total of 234 protein levels (17.1%) were significantly associated with global DTI measures. Of these, 29.9% was unique for FA, and 29.9% for MD, while levels of the remaining proteins were associated with both measures (WM-generic proteins). WM-generic proteins were mostly enriched for pathways related to lipid metabolism and in endothelial cells, whereas proteins specific to FA were mostly related to blood coagulation and enriched in astrocytes and those specific to MD were mainly associated with processes related to actin filaments and enriched in oligodendrocytes. When looking at the interaction with amyloid status, both global FA and MD alterations in A+ participants were associated with biological processes of axonogenesis and synaptic plasticity. Regional analysis revealed distinct proteomic profiles associated with variations in regional FA and MD, with processes linked to synaptic plasticity specifically related to integrity of limbic fibers.

**Discussion:**

Loss of WM integrity in the very early stages of AD seems to be related to alterations in biological processes associated with neuronal plasticity and oligodendrocyte integrity. Our findings provide new insights into the distinct biological mechanisms regulating WM integrity and its relationship with AD pathology.

## Introduction

Alzheimer disease (AD) begins years before dementia develops, with the accumulation of amyloid beta into plaques while cognition remains intact.^1^ This process can take up to 15 years before clinical symptoms emerge. During this period, amyloid plaques alter neuronal connectivity by impairing synaptic plasticity and affecting brain myelination.^2^ While extensive research has primarily focused on the detrimental convergence of AD pathology on neurons, emerging evidence has proposed a central role of white matter (WM) alterations in driving the disease.^3^ AD pathologic changes may in fact contribute to long-range WM disconnection, alterations of neuronal communication, and brain network dysfunction, ultimately playing a central role in cognitive decline.^4^ Improved understanding of the molecular processes involved in the decline of WM brain connectivity in early AD pathogenesis is important for developing novel secondary prevention therapies.

Diffusion tensor imaging (DTI) is an MRI technique that allows for in vivo characterization of WM connection integrity,^5^ using scalars such as fractional anisotropy (FA) and mean diffusivity (MD). FA has been hypothesized to capture general WM microstructural changes, while MD has been hypothesized to reflect cellularity, edema, and necrosis.^6^ Still, these metrics often strongly correlate, and it remains unknown to what extent they may reflect alterations in specific molecular processes. Results from recent DTI studies suggest that WM may be affected early on in the AD process, with widespread WM alterations already observed in cognitively unimpaired older individuals with initial amyloid deposition, revealing informative spatial gradients that vary depending on the underlying pathology.^7^ Improved understanding of their biological determinants is crucial to characterize early pathophysiologic mechanisms of neurodegenerative disease.

Animal and human studies have indicated that several biological processes may underlie alterations in WM integrity during normal aging and early AD. Age-related oligodendrocyte dysfunction, due to iron overload, oxidative stress, or endothelial dysfunction pathways, may promote demyelination and axonal loss in the initial stages of AD.^8^ This initial demyelination is believed to interact with lipid and cholesterol dysmetabolism to worsen amyloid pathology.^9^ Myelin damage can in turn promote the activation of inflammatory and immune pathways, also observable at the level of the WM with both postmortem^10^ and DTI in vivo studies.^11^ Astrocyte dysfunction and blood-brain barrier impairments could both enhance neuroinflammatory response and impede amyloid vascular clearance through glymphatic dysfunction.^12^ For a long time, such processes were difficult to study in detail in patients. However, recent advances in proteomics technologies such as mass spectrometry have made it possible to simultaneously measure the level of thousands of proteins from CSF,^13^ thus allowing the examination of in vivo biological processes such as lipid dysmetabolism, synaptic plasticity, and immune activation. Proteomic analyses have provided valuable insights into the biological processes underlying AD proteinopathies, revealing distinct mechanisms at different disease stages.^14^ Furthermore, individual proteomic profiles have enabled the identification of distinct disease subtypes, potentially reflecting different primary disease mechanisms and contributing to the observed heterogeneity of AD.^15^ However, little evidence exists about proteomics profiles that regulate WM integrity in the earliest disease stages, when several biological processes may contribute to initial WM degeneration.

We hypothesized that processes related to loss of axonal integrity as measured with DTI may be reflected by changes in CSF protein levels. Furthermore, we hypothesized that if FA and MD capture different aspects of axonal integrity, these metrics would show unique associations with proteins involved in distinct molecular pathways and preferentially expressed in specific cell types. For example, FA might be related to processes regulating myelination, but also vascular and astrocytic processes, whereas MD may more closely reflect axonal and cytoskeletal pathways, including microtubule organization, axonal transport, and structural maintenance. Finally, we hypothesized that proteins related to synaptic plasticity would be particularly associated with DTI metrics in participants with early amyloid deposition. In this study, we tested these hypotheses in older individuals without dementia who had DTI and CSF proteomic data available. We first investigated which proteins' CSF levels were associated with global FA and MD and then examined whether such associations were present in specific regions along major WM tracts. Finally, we tested whether the estimated relationships were dependent on amyloid status as an indication of early AD pathology.

## Methods

### Study Participants

For this study, we selected individuals who had CSF proteomics and DTI data available from 2 studies performed at the Amsterdam Alzheimer Center under similar protocols (i.e., European Medical Information Framework for AD [EMIF-AD preclinAD]^16^ and Amsterdam site participants who coenrolled in the ADC Biobank and the European Prevention of Alzheimer Dementia [EPAD] study^17^), who provided written informed consent to use their data and biospecimens for research purposes. Inclusion criteria in the EMIF-AD preclinAD cohort were age older than 60 years, a delayed recall score of >−1.5 SD of age-adjusted normative data on the Consortium to Establish a Registry for Alzheimer's Disease 10-word list,^18^ a global Clinical Dementia Rating (CDR) score of 0, a Telephone Interview for Cognitive Status modified score of 23 or higher,^19^ and a 15-item Geriatric Depression Scale score of <11.^20^ Eligibility criteria in the EPAD cohort were age older than 50 years, CDR score of <1, and no known diagnosis of dementia.^17^

### Standard Protocol Approvals, Registrations, and Patient Consents

All participants included in this study provided written informed consent, and ethical approval was obtained from ethics committees local to each study site.

### Diffusion MRI Acquisition and Processing

Details of diffusion MRI acquisition parameters are given in previous publications.^16,21^ In brief, in the EMIF-AD preclinAD cohort, diffusion images were acquired with a Philips 3T Achieva scanner (echo-planar imaging [EPI] sequence repetition time [TR] = 7,517 milliseconds, echo time [TE] = 92 milliseconds). In the EPAD participants, diffusion MRI (dMRI) scans were acquired with a Philips 3T Ingenuity MRI scanner (EPI sequence TR = 6,836 milliseconds, TE = 70 milliseconds). In both cohorts, diffusion gradients were collected over 32 noncollinear directions (*b* = 1,000 s/mm^2^), and 1 additional scan per participant with no diffusion weighting (*b* = 0 s/mm^2^) was acquired.

Preprocessing was performed using QSIPrep 0.19.0, which is based on Nipype 1.8.6.^22^ Preprocessing included Marchenko-Pastur principal component analysis denoising as implemented in MRtrix3's dwidenoise,^23^ B1 field inhomogeneity correction using dwibiascorrect from MRtrix3 with the N4 algorithm,^24^ and estimation and correction of head motion and eddy current–induced distortions using the FSL Eddy tool.^25^ A deformation field was estimated using a fieldmap-less approach and used to correct for EPI susceptibility.^26^ Preprocessed dMRI scans were fed to a brain extraction algorithm, and FSL DTIFIT was then used to fit the diffusion tensor model to the data and produce DTI scalar maps, that is, FA and MD.

To compute global and regional DTI values, we used tract-based spatial statistics (TBSS) from FSL.^27^ FA images, obtained after tensor fitting, were aligned to a common space using nonlinear registration. Aligned FA maps were visually quality checked. Next, the mean FA image was thinned to create a mean FA skeleton representing the center of all tracts common to the group. MD images were then aligned to the same space using the computed registrations. The derived FA skeleton was used to compute mean FA and MD as a global metric of WM integrity. Skeletonized FA and MD values were further averaged within 12 selected skeletonized tracts defined based on the JHU ICBM-DTI-81 atlas,^28^ subdivided into commissural (splenium, body, and genu of corpus callosum [CC]), associative (superior longitudinal, superior fronto-occipital, and inferior longitudinal [IL] fasciculus), limbic (fornix and cingulum), and projection (anterior and posterior corona radiata [PCR], anterior and posterior limb of the internal capsule [PLIC]) tracts. DTI scalars (global and regional values) were harmonized across cohorts using NeuroCombat,^29^ while keeping the variance explained by age, sex, and amyloid status to account for acquisition protocol differences and maintain biological variance.^29^ TBSS skeleton and the used skeletonized atlas are shown in eFigure 1. NeuroCombat harmonization results are shown in eFigure 2.

### CSF Analysis

CSF was obtained through lumbar puncture. CSF sample collection, processing, and storage at the Alzheimer Center Biobank in the Neurochemistry Laboratory of the Department of Laboratory Medicine were performed according to the international guidelines.^30^ CSF levels of amyloid-beta 1–42 (Aβ1‐42) were measured with Elecsys (EPAD) or ADxNeurosciences/EUROIMMUNE ELISA (EMIF-AD preclinAD). Participants were classified as amyloid negative (A−) or positive (A+) according to assay-specific cutoffs.^31,32^ Untargeted tandem mass-mass spec proteomics with a 16-plex was analyzed by liquid chromatography-tandem mass spectrometry as previously described in detail.^15^ A total of 3,863 unique proteins were identified, of which 1,372 proteins were observed across all individuals and included in further analysis. Before statistical analyses, we first normalized the protein levels to account for potential technical variation between the 16-plex measures. In brief, we used the internal reference scaling normalization procedure,^33^ adapted to scale according to the median instead of the total sum, to reduce the influence of outlier values and ensure robust normalization across samples. Subsequently, we further standardized protein levels to the mean and standard deviation of individuals with intact cognition (CDR = 0) and normal AD CSF markers, facilitating the comparison of effect sizes between proteins.

### Statistical Analysis and Gene-Set Enrichment Analysis

Linear models were used to investigate the association between protein levels and both global and regional FA and MD values. Models also included an interaction term between protein levels and amyloid status (A−/A+). Models were corrected for age and sex. Because we were interested in understanding pathways associated with each of the outcome measures (global and regional FA and MD), we selected proteins according to a lenient significance threshold of *p* < 0.05 for subsequent pathway analyses. We used the function enrichGO from the R package “clusterProfiler”^34^ to perform gene-set enrichment analysis (GSEA), with Gene Ontology^35^ as a reference gene source for functional profiling. *p* Values for pathways were corrected for multiple testing with the false discovery rate procedure, and we considered significance for false discovery rate values below 0.05. GSEA was performed for proteins associated with each outcome measure (global and regional DTI scalars) separately. GSEA results were compared using the compareCluster function from the same package and visualized through emapplot. Cluster comparison was performed between global FA and MD, and between regional FA and MD values. Sensitivity analyses were performed to evaluate the influence of correcting for cardiovascular (hypertension, diabetes, and hypercholesterolemia) and cerebrovascular (WM hyperintensities) factors in the linear models (eFigures 3 and 4). Furthermore, we repeated the analyses within each cohort to evaluate possible cohort-specific effects.

### Cell-Type Enrichment Analysis

For proteins associated with global DTI scalars, we further performed a cell-type enrichment analysis. We annotated proteins according to cell-type specificity using the Human Protein Atlas and the RNA-seq Barres database.^36^ We specifically examined cell-type profiles of proteins that were enriched in both global FA and MD, only in FA, and only in MD.

### Data Availability

All mass spectrometry data part of this study with accompanying demographical information are available through the Alzheimer's Disease Data Initiative workbench (fair.addi.ad-datainitiative.org/#/data/datasets/five_csf_proteomic_subtypes_in_ad). The imaging data used for this work are openly available on request (amypad.eu/).

## Results

### Participant Characteristics

Baseline demographics and clinical characteristics are listed in Table 1, stratified by amyloid status. In total, 96 participants were included in the study. Based on CSF Aβ1-42 levels, 43% were defined as having abnormal amyloid levels. The mean age was 67.82 (6.93) years, 43 (45%) were male, and 43% of the included participants had at least 1 ε4 allele in the *APOE* gene.

**Table 1:** Cohort Characteristics

	Overall (N = 96)	A− (n = 54)	A+ (n = 42)
Age, y, mean (SD)	67.82 (6.93)	66.01 (5.76)	70.15 (7.66)
Sex, male, n (%)	43 (44.8)	25 (46.3)	18 (42.9)
MMSE score, mean (SD)	28.78 (1.45)	29.00 (1.21)	28.49 (1.68)
*APOE* carrier, n (%)	43 (45.3)	16 (29.6)	27 (65.9)

Abbreviations: A− = amyloid negative; A+ = amyloid positive; MMSE = Mini-Mental State Evaluation.

### Proteomics Signatures of Global WM Integrity

**Figure 1:**
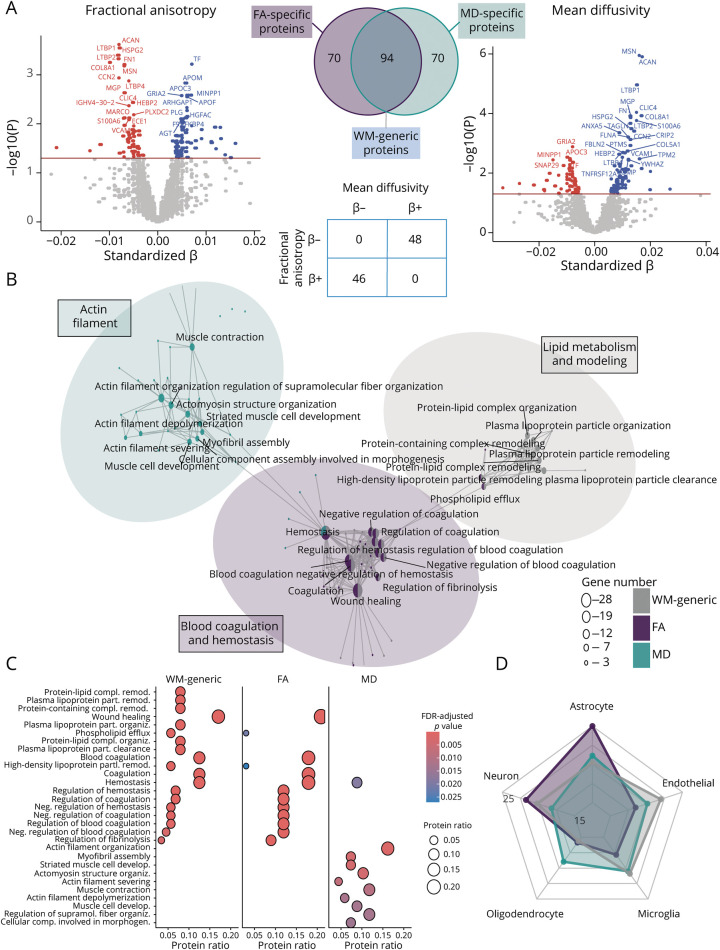
CSF Proteomics Profiles Regulating Global FA and MD (A) Proteins associated with FA (left) and MD (right) are shown in the volcano plots. Overlapping and specific protein numbers for each DTI metric are shown in the Venn diagram and contingency matrix. (B) Results of the gene-set enrichment analysis for WM-generic, FA-specific, and MD-specific protein association. (C) Dot plot showing the most enriched pathways for WM-generic, FA-specific, and MD-specific proteins. (D) Results of the cell-type enrichment analysis. DTI = diffusion tensor imaging; FA = fractional anisotropy; MD = mean diffusivity; WM = white matter.

Across the group, higher global FA values were strongly related to lower MD values (*r* = −0.88, *p* < 0.001), as expected. Next, we tested associations of these values with CSF protein levels and observed that 234 proteins were related to either or both measures (Figure 1A). Specifically, CSF levels of 94 proteins showed a significant association with both global FA and MD, hereafter referred to as *WM-generic* proteins. Of these, 46 showed a positive association with FA and negative association with MD, while the remaining 48 had an opposite trend (Figure 1A). These WM-generic proteins were mostly enriched for genetic pathways related to 2 clusters of biological processes (Figure 1, B and C). The first cluster was linked to pathways of lipid metabolism, lipoprotein organization and modeling, and cholesterol efflux, while the second cluster was linked to pathways of regulation of blood coagulation and hemostasis. Moreover, these proteins were more prominently enriched in endothelial cells (Figure 1D). We also found CSF levels of 70 proteins to be specifically related to FA values, that is, FA-specific proteins. These proteins were also found to be associated with pathways of blood coagulation and hemostasis (Figure 1, B and C) and were mostly enriched in astrocytes and in neurons (Figure 1D). Finally, CSF levels of 70 proteins were distinctively related to MD values, that is, MD-specific proteins. These proteins were specifically associated with a third cluster of pathways related to actin filament organization and development, as well as cell assembly (Figure 1, B and C), and were found to be mostly expressed in oligodendrocytes (Figure 1D). When repeating the analysis within each cohort separately, protein effect sizes (betas) closely overlapped with those from the full models, suggesting robust effects across cohorts (eFigure 2B). High protein-beta correlations were also observed when correcting for both cardiovascular (hypertension, diabetes, and hypercholesterolemia) and cerebrovascular (WM hyperintensities) factors in the linear models (eFigures 3 and 4, and eTable 1).

### Amyloid-Dependent Association of Proteomics and Global WM Integrity

**Figure 2:**
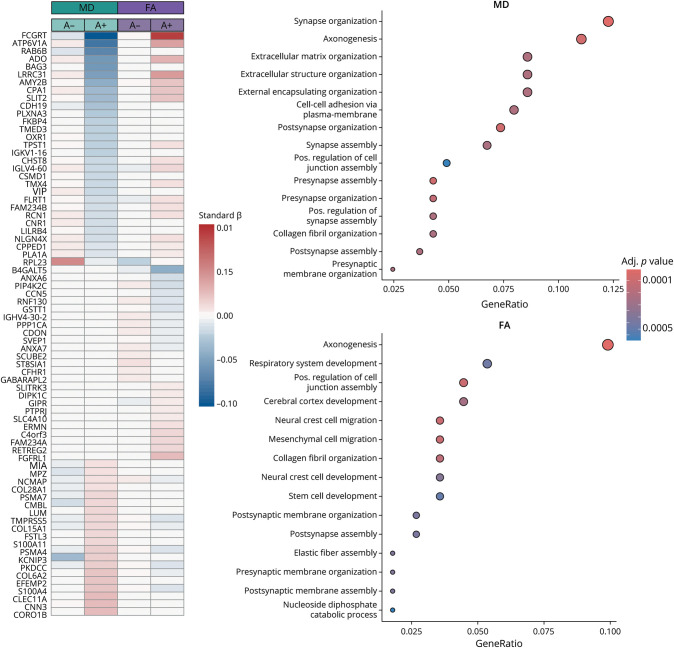
Interaction of Amyloid Status and Proteomics Driving White Matter Integrity On the left, the heat map demonstrates the 50 proteins showing the strongest interaction with amyloid status in predicting WM integrity. Group-specific (A−/A+) standardized beta values are displayed per each DTI metric. On the right, the dot plots show the 15 most enriched pathways. DTI = diffusion tensor imaging; FA = fractional anisotropy; MD = mean diffusivity; WM = white matter.

We then examined whether proteins were associated with WM integrity global metrics in an amyloid-dependent manner. In total, 228 proteins showed significant interaction with amyloid status in predicting global DTI metrics. Of them, 52 were uniquely associated with global FA, 104 were uniquely associated with global MD, and 72 were associated with both FA and MD. Figure 2A shows the 50 proteins that were most strongly associated with FA and MD in interaction with amyloid. The full list and coefficients are provided in eTables 2 and 3. Generally, these proteins showed a stronger association with DTI metrics in A+ individuals compared with A− individuals. When performing GSEA, we found that all these proteins (for both FA and MD) were related to biological processes linked to axonogenesis, synaptic plasticity and organization, and cell migration (Figure 2).

### Proteomics Signature of Regional Variations in WM Integrity

**Figure 3:**
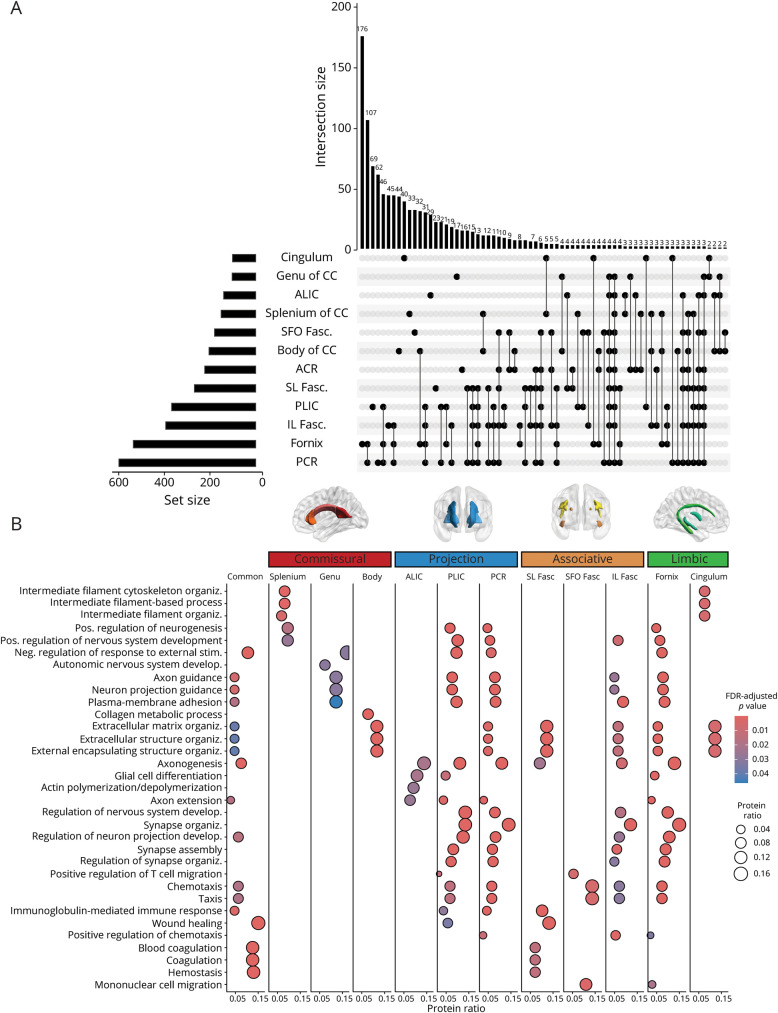
Proteomic Signatures of Regional Variations in WM Integrity (A) Upset plot showing the number of proteins associated with each WM tract of interest and the overlap between tracts. (B) Dot plot showing most enriched biological pathways per tract of interest. WM = white matter.

Finally, we studied whether the associations of protein levels with FA and MD values were specific for particular anatomic regions of the WM. Table 2 presents the number of total, upregulated, and downregulated significant proteins for each of the regional DTI metrics. We observed distinct clusters of biological processes contributing to regional variations in FA. Figure 3 shows the proteins uniquely contributing to each region and the (70 largest) intersections between regions. The region of the fornix showed the strongest association with CSF protein levels, with 655 and 670 significantly associated with FA and MD in this region, respectively. A sensitivity analysis of the fornix results is illustrated in eFigure 5 (skeletonized fornix mask used), eFigure 6 (replication using a different fornix mask), and eTable 4 (coefficients of linear models). These proteins were mostly enriched for pathways linked to synaptic organization, plasticity, and axon extension, as well as neurodevelopmental pathways. A similar profile (synaptic + development) was also observed for the IL fasciculus, PCR, and PLIC. A different set of proteins were found to be related to commissural fibers, mostly linked to neurodevelopmental biological processes. The splenium of the CC, for example, was enriched for cytoskeleton organization, neurogenesis, and nervous system development pathways, whereas the genu was enriched for axon and neuron projection guidance, but also for response to external stimuli. When looking at proteins commonly associated across regions, these were mostly enriched for biological processes of blood coagulation and hemostasis, aligning with global FA results.

**Table 2:** Proteins Significantly Associated With Regional DTI Metrics

	FA			MD		
	Total	Positive β	Negative β	Total	Positive β	Negative β
CC (body)	312	165	147	175	101	74
CC (splenium)	187	106	81	208	118	90
CC (genu)	163	94	69	146	72	74
Cingulum	143	94	49	131	70	61
Fornix	655	504	151	670	180	490
IL fasciculus	315	146	169	206	140	66
SL fasciculus	199	101	98	208	143	65
SFO fasciculus	160	82	78	255	164	91
ALIC	126	48	78	224	149	75
PLIC	225	99	126	158	98	60
ACR	206	106	100	261	162	99
PCR	519	169	350	253	152	101

Abbreviations: ACR = anterior corona radiata; ALIC = anterior limb of internal capsule; CC = corpus callosum; DTI = diffusion tensor imaging; IL = inferior longitudinal; PCR = posterior corona radiata; PLIC = posterior limb of internal capsule; SFO = superior fronto-occipital; SL = superior longitudinal.

## Discussion

We found that FA and MD present only partially overlapping proteomic signatures, suggesting that they capture different (or specific) underlying pathophysiologic processes involved in loss of WM integrity. Moreover, we observed that with initial amyloid deposition, the activation of biological pathways linked to synaptic plasticity and axonogenesis is related to WM damage, and that this might be more evident in limbic and posterior WM tracts.

Previous studies on WM integrity and protein levels mainly focused on targeted CSF or plasma markers in early AD, particularly Aβ1–42, phosphorylated tau 181, and total Tau.^37^ These studies suggested that more abnormal levels of CSF Aβ1–42 and phosphorylated tau 181 were related to reductions in WM integrity already in cognitively unimpaired individuals,^7^ with stronger effects in limbic tracts, such as the fornix. Other studies have evaluated the association between neurofilament light chain, a protein linked to axonal damage across neurodegenerative disease, and microstructural WM alterations.^38^ Our results, based on an untargeted mass-mass spec approach, further extend the existing literature by showing that specific proteomic signatures are related to different DTI-derived WM metrics. While FA and MD are strongly correlated, as we also observed, they have been hypothesized to reflect different biological properties. We now find that while approximately one-third of the proteins were related to both metrics, a considerable portion was specifically associated with either FA or MD. Specifically, both FA and MD were associated with lipid metabolism, in line with the known role of lipids in WM function, as observed in previous studies using fluids,^39^ genetics,^40^ and histopathologic^41^ data from humans, reinforcing the idea that these markers reflect myelin-related processes.^4^ In addition, FA also showed specific associations with processes related to vascular and coagulation factors, whereas MD was more specifically associated with axonal and synaptic structural (actin filament)–related processes. These results are supported by neurobiological evidence from animal studies. For instance, neuropathologic observations in neonatal piglets^42^ found that FA from in vivo diffusion MRI correlated with swollen astrocytes and myelin loss. In the same study, MD was also specifically associated with degenerating myelinating oligodendrocytes, similar to our results. Additional studies in rats and mice have further highlighted the influence of astrocytes on FA under various conditions.^43^ In this study, we demonstrated these associations in vivo in humans by using proteomics data. Taken together, these results are in line with the idea of FA being a general microstructural index, possibly reflecting vascular-mediated injury processes and MD being related to cellularity, membrane density, and cytoskeletal alterations,^6^ suggesting that these biomarkers capture partially different aspects of WM damage. However, it is important to note that CSF proteomics lack both regional as well as cell-type specificity, and some of the measured proteins could be expressed by multiple cell types. This limits the interpretability of the cell-type enrichment results and underscores the need for further validation.

Furthermore, our findings indicate that different WM tracts are associated with diverse underlying pathophysiologic processes, reinforcing the notion that spatial patterns of WM disruptions are largely driven by distinct biological dysfunctions.^7^ However, no study has previously examined the in vivo association between several biological processes, as measured by CSF proteomics, and WM integrity. Previous work using proteomics from ex vivo WM samples revealed 7 functional groups of WM-expressing proteins, including metabolic proteins, structural proteins, proteins involved in signal transduction, blood proteins, stress-related proteins, and proteins involved in the ubiquitin-mediated proteolysis.^44^ Another study on small vessel disease, which can co-occur with AD, previously reported that WM lesion volume measured from fluid-attenuated inversion recovery MRI data in vivo were associated with CSF proteomic signatures that included proteins linked to metabolism and enriched in endothelial and smooth muscle cells with early damage.^45^ This study further observed that inflammation-related proteins expressed in microglial cells, such as Integrin Subunit Alpha M and interleukin 15, were associated with the progression of WM lesions into more advanced stages. Building on these results, our findings provide new insights into the molecular mechanisms contributing to WM alterations by linking large-scale proteomics data to DTI-derived metrics. Specifically, our findings highlight associations between DTI indices and pathways related to blood coagulation, lipid metabolism, and actin filament dynamics. Posterior and limbic tracts were further enriched for synaptic plasticity pathways, while commissural fibers were related to neurogenesis and axonogenesis pathways. These pathways may point to key mechanisms driving early regional WM alterations, such as vascular contributions, membrane remodeling, and cytoskeletal changes. Furthermore, incorporating proteomic profiles into clinical or trial settings may enhance early detection of WM damage, stratify patients based on underlying mechanisms, and refine interventions tailored to specific molecular pathways.

Another finding in our study is that we observed specific proteomic signatures related to worse WM measures in individuals with abnormal amyloid only. In these individuals, proteins related to synaptic plasticity, neuronal development, and axonogenesis were associated with WM integrity, which was not observed in individuals with a normal amyloid status. The observed interaction between amyloid status and WM-associated proteins indicates that the relationship of protein levels measured in CSF with FA and MD is dependent on amyloid status, possibly reflecting complex, bidirectional pathophysiologic processes that cannot be directly tested in our cross-sectional study. On one hand, disturbed synaptic connectivity due to amyloid plaque deposition may disrupt axonal connectivity as well, possibly by interfering with lipid metabolism and myelin repair processes^4,8^ and/or through impaired glial cell functioning.^9^ Animal studies have shown that presynaptic and postsynaptic accumulation of amyloid promote alterations of synaptic excitatory transmission, subsequently leading to axonal transport defects and axonal damage.^46^ Of interest, the link between local synaptic and axonal transport perturbations might be partially mediated by tau phosphorylation,^47^ although this mechanism could not be assessed in this study. On the other hand, recent experimental evidence demonstrates that myelin dysfunction can act as an upstream driver of amyloid pathology.^48^ This suggests the existence of a vicious cycle where amyloid accumulation compromises local synapses, disturbing axonal transport, thus creating axonal swellings and myelin dysfunctions, which in turn can promote the formation of Aβ plaques. This signature also included proteins that have been related to oligodendrocytes and astrocytes, which have previously been implied in neurodegenerative processes in AD pathogenesis, and that play a key role in WM architecture and function,^3^ posing WM dysfunction as a central driver of AD progression.

It is important to note that a cluster of pathways linked to synaptic plasticity and neural development was also found to be associated with the regional distribution of FA and MD, specifically in limbic and posterior projections tracts, and most strongly in the fornix. These observations likely reflect several converging mechanisms. First, the fornix is an important tract of the limbic system, connecting regions that play a central role in memory functions.^49^ Moreover, this observation is also consistent with previous literature demonstrating a gradient of WM regional vulnerability to distinct pathophysiologic processes,^7^ which follows an inverse myelination pattern, where late-myelinating tracts—like the fornix—show early susceptibility to pathologic processes.^50^ This molecular-structural interaction might precede typical mediotemporal neurodegenerative processes observed in later disease stages, but future longitudinal studies using both in vivo and ex vivo MRI and histopathology are needed to further investigate this question. Moreover, the results involving the fornix should be interpreted with caution because this tract is small, CSF-adjacent, and anatomically variable. The use of template-based, skeletonized regions of interest may be susceptible to misregistration and partial-volume contamination. As such, our fornix findings are best viewed as exploratory and hypothesis-generating rather than definitive.

Although we used a unique design to study molecular processes underlying MRI-based metrics using in vivo large-scale CSF proteomics, a potential limitation may be that we were limited to 96 participants included in this study and lack a validation cohort. However, few cohorts currently have both DTI acquisitions and untargeted tandem mass-mass spec proteomics data, and few studies have looked at the association of these 2 types of data. Participants included were highly educated, and future studies should address the generalizability of our results to the general population. Moreover, our data only included single-shell DTI acquisition, thus limiting our ability to compute more advanced metrics, such as free water–corrected scalar values. Still, we observed significant associations with both FA and MD, suggesting that more advanced DTI may pick up other aspects of WM integrity that could provide more insight into the structural changes underlying cognitive decline in AD. Although we included CSF tau levels and tangle burden was likely limited, given that most participants had intact cognition, we could not directly control for individual differences in tangle burden, because measures specific to it, such as tau PET, were not available. Future studies should aim to include such measures to understand to what extent biological staging of AD may influence the relationship between CSF protein levels and MD/FA values on MRI. Furthermore, our design was cross-sectional, and longitudinal studies are required to further investigate which molecular processes change together with loss of integrity and cognitive function in the same individuals. Finally, as already mentioned, CSF proteomics does not have any cell-type or regional specificity, and future studies using tissue-specific proteomics data should aim at confirming our results.

It is important to also note that several factors, related to either vascular or neurodegenerative processes (or to both), can regulate WM health in late life. The aim of this study was not to disentangle the relative contributions of vascular vs neurodegenerative processes to WM damage, but rather to describe biological processes that regulate WM health in older age. We found that CSF protein levels indicative of blood brain barrier dysfunction were related to FA, further supporting the idea that vascular alterations may contribute to the changes in DTI-based values observed in AD. For this reason, we performed sensitivity analysis to understand to what extent these results were driven by cerebrovascular and cardiovascular factors and found that our results were mostly confirmed when correcting for these factors in the analysis. However, future studies should more precisely investigate such mechanisms examining, for example, possible interactions and independent effects and using other potential measurements such as arterial spin labeling to understand the potential vascular contribution to alterations in DTI-derived measurements. In summary, we found that MRI-based measures of FA and MD capture loss of WM integrity in early AD and that these metrics are able to capture at least partially different facets of the AD pathologic cascade and of underlying molecular processes, showing a regional gradient of biological susceptibility.

## Glossary

Aβ1‐42: amyloid-beta 1–42
AD: Alzheimer disease
CC: corpus callosum
CDR: Clinical Dementia Rating
dMRI: diffusion MRI
DTI: diffusion tensor imaging
EMIF-AD: European Medical Information Framework for AD
EPAD: European Prevention of Alzheimer Dementia
EPI: echo-planar imaging
GSEA: gene-set enrichment analysis
FA: fractional anisotropy
IL: inferior longitudinal
MD: mean diffusivity
PCR: posterior corona radiata
PLIC: posterior limb of internal capsule
TBSS: tract-based spatial statistics
TE: echo time
TR: repetition time
WM: white matter
